# Cytotoxic and HIV-1 enzyme inhibitory activities of Red Sea marine organisms

**DOI:** 10.1186/1472-6882-14-77

**Published:** 2014-02-25

**Authors:** Mona S Ellithey, Namrita Lall, Ahmed A Hussein, Debra Meyer

**Affiliations:** 1 Department of Biochemistry, University of Pretoria, Pretoria, 0002, South Africa; 2 Department of Plant Science, University of Pretoria, Pretoria, 0002, South Africa; 3 Department of Chemistry, University of the Western Cape, Private Bag X17, Belleville, 7535, South Africa

**Keywords:** Red Sea, Marine organisms, Cytotoxicity, HIV‒1 protease and HIV-1 reverse transcriptase

## Abstract

**Background:**

Cancer and HIV/AIDS are two of the greatest public health and humanitarian challenges facing the world today. Infection with HIV not only weakens the immune system leading to AIDS and increasing the risk of opportunistic infections, but also increases the risk of several types of cancer. The enormous biodiversity of marine habitats is mirrored by the molecular diversity of secondary metabolites found in marine animals, plants and microbes which is why this work was designed to assess the anti-HIV and cytotoxic activities of some marine organisms of the Red Sea.

**Methods:**

The lipophilic fractions of methanolic extracts of thirteen marine organisms collected from the Red Sea (Egypt) were screened for cytotoxicity against two human cancer cell lines; leukaemia (U937) and cervical cancer (HeLa) cells. African green monkey kidney cells (Vero) were used as normal non-malignant control cells. The extracts were also tested for their inhibitory activity against HIV-1 enzymes, reverse transcriptase (RT) and protease (PR).

**Results:**

Cytotoxicity results showed strong activity of the Cnidarian *Litophyton arboreum* against U-937 (IC_50;_ 6.5 μg/ml ±2.3) with a selectivity index (SI) of 6.45, while the Cnidarian *Sarcophyton trochliophorum* showed strong activity against HeLa cells (IC_50;_ 5.2 μg/ml ±1.2) with an SI of 2.09. Other species showed moderate to weak cytotoxicity against both cell lines. Two extracts showed potent inhibitory activity against HIV-1 protease; these were the Cnidarian jelly fish *Cassiopia andromeda* (IC_50;_ 0.84 μg/ml ±0.05) and the red algae *Galaxura filamentosa* (2.6 μg/ml ±1.29). It is interesting to note that the most active extracts against HIV-1 PR, *C. andromeda* and *G. filamentosa* showed no cytotoxicity in the three cell lines at the highest concentration tested (100 μg/ml).

**Conclusion:**

The strong cytotoxicity of the soft corals *L. arboreum* and *S. trochliophorum* as well as the anti-PR activity of the jelly fish *C. andromeda* and the red algae *G. filamentosa* suggests the medicinal potential of crude extracts of these marine organisms.

## Background

Life-threatening illnesses such as cancer and the acquired immunodeficiency syndrome (AIDS) presents patients and their families with considerable burdens. For many sufferers both cancer and AIDS have evolved from acutely terminal conditions into chronic illnesses characterized by complex psychosocial and physical issues.

The number of people living with HIV/AIDS in 2010 was estimated to be around 34 million [1]. On the other hand a total of 1,638,910 new cancer cases and 577,190 deaths from cancer were determined to have occurred in the United States in 2012 [2]. Lower CD4 (white blood cell) counts and a weakened immune system are risk factors for pre-cervical and cervical cancer. Additionally, women with HIV are more likely to have a recurrence of pre-cervical cancer and antiretroviral therapy did not protect HIV-positive women from the development of pre-cervical cancer [3-5].

In order to combat both cancer and HIV/AIDS, colossal amounts of money, manpower, time and energy have been dedicated to research on novel compounds which can be developed as therapeutic agents [2]. Anti-cancer drugs and HIV treatment are related since nucleotide analogues can be used for the treatment of both diseases. Using natural products to manufacture drugs is an ancient and well established practice that has yielded familiar products such as morphine, digitalis, penicillin, and aspirin [6].

Mankind has known for thousands of years that marine organisms contain substances capable of potent biological activity which has recently also been demonstrated against different types of cancer and HIV/AIDS [7]. Natural product screening has been a component of drug-lead development for the better part of 20 years. Novel pharmaceuticals, such as taxol, have been discovered through the screening of extracts from plants, microorganisms and marine organisms [8], while *in vitro* inhibition of key enzymes of the viral life cycle serves as a first step in HIV/AIDS drug development.

The studies on the antiviral activities of marine natural products are presently attracting more and more attention worldwide. Marine derived compounds have been shown to have a variety of bioactivities such as antiviral, anticoagulant, antioxidant and other medicinal properties [9]. HIV-1 enzymes RT and PR were identified early on as potential drug targets. The discovery and development of inhibitors of these enzymes are an unqualified success of modern pharmacology and structural biology [10]. The emergence of drug-induced mutations of HIV-1 enzymes leads to rapid loss of the potency of existing drugs and the need to develop new candidates [11].

The Red sea represents one of the most promising areas as a source of medicinal natural products. Most of the investigations into the biological activities of organisms from the Red Sea was conducted either in the northern part of gulf Aqaba [12,13] or the southern part of the sea and reported on the organisms’ free radical scavenging and cancer growth inhibition activities [14-16]. These areas are subject to tourism and human impact, which can extensively affect the marine community. As reported by “Zalul,” an Israeli environmental lobby group, ‘over the past five years the most northerly part of the Red Sea coral reef is now 70% dead’ [17].

The study area investigated in the current report, Sharm El-Sheikh, is located in the connection point of the south Suez Gulf and south Aqaba Gulf. Most of this region is protected by the Egyptian Ministry of State For Environmental Affairs. Despite its high water quality, there are no published reports for the biological activities of marine organisms from Sharm El-Sheikh which prompted the present investigation.

This study was designed to investigate the potential cytotoxic and anti-HIV activities of thirteen marine organisms collected from Sharm El-Sheikh, Red Sea (Egypt).

## Methods

### Marine organisms

Thirteen of the most abundant marine organisms from different families were collected from Nabq and Ras Mohammed protected areas (Sharm el-Sheikh, Red Sea, Egypt) in the period between March and April 2010. Fifty gram (50 g) of each sample was collected according to the sampling and preservation protocol of Kathrina Fabricius [18]. The material was collected and identified by Mona Ellithey, co-author of this article, using Red sea invertebrates’ reference guide [19] which also provided detailed information specimen classification as indicated per organism below. A voucher specimen has been deposited in the Marine Natural products laboratory, NRC (Egypt) with deposition number 10/1:13. Samples were collected from different marine environments. Two sponges; *Spongia officinalis* (Linnaeus, 1759), *Haliclona rubens* (sensu Duchassaing & Michelotti, 1864) and the jelly fish *Cassiopea andromeda* (Forskål, 1775) were collected from 0.6-1 m depth of mangrove swamps. The sandy bottom was used for collection of the algae *Cymodocea rotundata* (Ehrenberg & Hemprich ex Ascherson 1870), *Caulerpa prolifera* (Forsskål J.V. Lamouroux 1809) and the gastropod *Bulla ampulla* (Linnaeus, 1758), from depths of 30-60 cm. From the stony bottom of the coral reef the algae *Galaxaura filamentosa* (W.R. Taylor 1945), the soft corals *Litophyton arboreum* (Forskål, 1775), *Sarcophyton trochliophorum* (Von Marenzeller, 1886), *Sinularia heterospiculata, Sinularia maxima, Sinularia polydactyla* (Verseveldt, J. 1976) and *Turbinaria turbinatea* (Linnaeus Kuntze 1898) were collected at depths of 1-3 m.

### Extract preparation

Fresh marine organisms (50 g each) were homogenized directly after collection with 150 ml of 90% methanol (Merck, Germany). After filtration the solvent was evaporated under reduced pressure using a rotary evaporator (Buchi, Switzerland) and the residues re-suspended in water and partitioned with ethyl acetate in order to get rid of the salts and the high molecular weight hydrophilic compounds. The lipophilic fractions obtained by ethyl acetate were dried out by rotavapor and were then tested for their *in vitro* cytotoxicity against two human cancer cell lines (U937) and (HeLa), and the African green monkey kidney cell line (Vero) as representative of a normal, non-cancerous cell line. The extracts were also tested for inhibitory activity in direct enzyme assays against HIV-1 reverse transcriptase and HIV-1 protease.

### Cell culture and cytotoxicity of extracts

Chemicals and reagents; all cell lines, media, trypsin-EDTA, fetal bovine serum (FBS) and antibiotics (penicillin, streptomycin and fungizone) were purchased from Highveld Biological (Pty) Ltd. (Modderfontein, Johannesburg, RSA).

The ethyl acetate extracts were each dissolved in DMSO to a stock solution of 20 mg/mL and added to the microtitre plate. Serial dilutions were made to range from a concentration of (400 μg/ml to 3.12 μg/ml) for each extract. The negative control wells included cells exposed to 2% DMSO. And the positive control Actinomycin D with concentrations ranging between 0.5 μg/mL and 0.002 μg/mL. The microtitre plate was incubated for a further 72 h. and were tested for their cytotoxic activity using the 2,3-bis-(2-methoxy-4-nitro- 5-sulfophenyl)-2H-tetrazolium-5-carboxanilide, Na2) (XTT) colorimetric assay. The assay is based on the ability of live cells to reduce the yellow water soluble XTT into an insoluble formazan product [20]. The marine extracts were first screened for their *in vitro* cytotoxicity at a concentration of 100 μg/ml against HeLa and U937 cells. Extracts which reduced > 50% of the cell proliferation of both cell lines were further tested at concentrations that ranged from (400 μg/ml to 3.12 μg/ml). A third non-cancerous cell line (Vero) was also included for the most active extracts in order to determine selectivity indices which represent the overall activity of the extract. Selectivity Indexes (SI) values were calculated as follows; the 50% inhibitory concentration (IC_50_) of the extract tested in Vero cell line was divided by the IC_50_ of the extract tested in a cancer cell. Higher SI values indicate the more selective extracts.

Cells were maintained in culture flasks in complete medium supplemented with 10% heat-inactivated FBS and antibiotic cocktail (100 U/mL penicillin, 100 g/L streptomycin and 250 g/L fungizone). Cells were cultured and maintained in a humidified atmosphere at 37°C and 5% CO_2_. Cytotoxicity was measured by the XTT method using the Cell Proliferation Kit II (F. Hoffmann-La Roche Ltd.). Cells (100 μl) were seeded (concentration 1 × 10^5^ cells/mL) into a microtitre plate and incubated for 24 h to allow the cells to adhere. Following the evaporation of ethyl acetate, the extract powder residue was dissolved in DMSO and then serially diluted (400 μg/ml to 3.12), added to the plates and incubated for 72 h. A positive control for cytotoxicity, actinomycin D and a negative control of cells with 2% DMSO were also included. After a 72 h incubation, XTT was added to a final concentration of 0.3 mg/mL and the cells incubated for 2–3 h. Absorbance of the developed colour was spectrophotometrically determined using a multi-well plate reader which measured the optical density at 450 nm with a reference wavelength of 690 nm. Mean IC_50_ is the concentration of extracts which reduces cell growth by 50% under the experimental conditions and is the average of at least three independent reproducible measurements. The IC_50_ values were performed using GraphPad Prism (San Diego, USA).

### HIV-1 direct enzyme assays

Reverse transcriptase (RT) inhibitory activity of the crude extracts against a purified recombinant HIV1-RT (Merck, Darmstadt, Germany) was determined by using the Roche Diagnostics (Mannheim, Germany) colorimtric kit. The assay was performed as previously described [21].

HIV-1 protease enzyme (Bachem Bioscience Inc. King of Prussia, PA, UK) and the substrate (a synthetic peptide that contains a cleavage site Tyr-Pro for HIV protease as well as two covalently modified amino acids for the detection of cleavage). The assay was performed according to procedures by Lam *et al.*[22] in black 96 well assay plates obtained from Corning Incorporated, (Corning, New York, USA). The fluorescence intensity was measured at an excitation wavelength of 355 nm and an emission wavelength of 460 nm using a synergy microplate spectrofluorometer (BioTek, Analytical & Diagnostic products, South Africa). Acetyl pepstatin (AP) was used as a positive control for HIV-1 PR inhibition. The blank treatment consisted of assay buffer with only the substrate and an untreated control of enzyme and substrate was also included. The percentage inhibition was calculated based on the formula:

$$\begin{array}{ll} 100 & –\left[(\mathrm{Test}\;\mathrm{reagent}\;\mathrm{RFU}–\mathrm{background}\;\mathrm{RFU}\right)/\\ & \;\left(\mathrm{untreated}\;\mathrm{control}\;\mathrm{RFU}–\mathrm{blank})\times 100\right] \end{array}$$

where RFU = relative fluorescence units.

All the marine organism materials were screened at 100 μg/ml for inhibition of HIV-1 enzymes. IC_50_ values of the most active extracts were calculated and compared to known HIV-1 PR and HIV-1 RT inhibitors. All the experiments were done 3 times in order to ensure the precision and accuracy of the data.

### Statistical analysis

One way ANOVA analysis was done followed by post hoc Tukey HSD tests to determine the significance of the extracts’ cytotoxicity and inhibitory activities. The statistical analysis was done using IBM SPSS version 19.

## Results and discussion

### Cytotoxicity of the extracts

**Figure 1:**
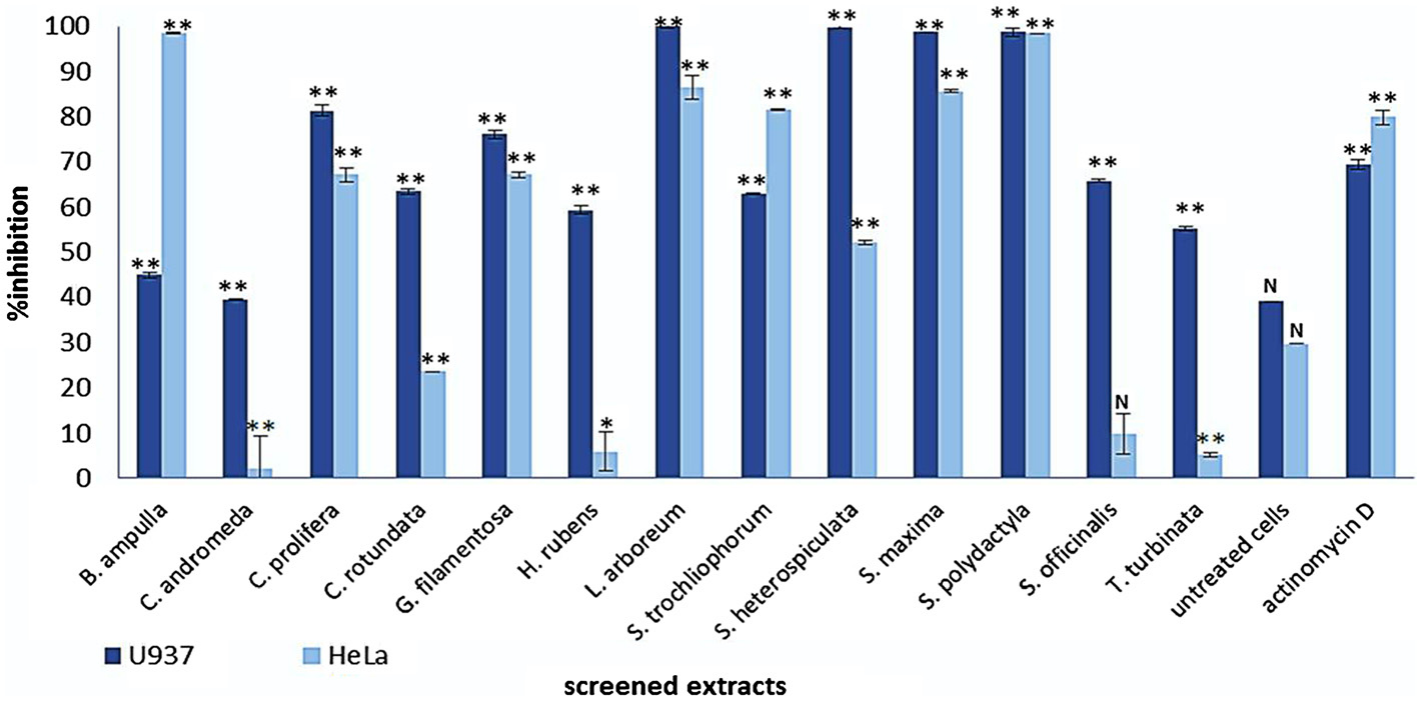
**Cytotoxicity of different marine extracts.** Samples were dissolved in DMSO and diluted with the medium to a final concentration of 100 μg/ml. Significant differences between the extracts and the DMSO control was calculated using IBM SPPS version 19. **P < 0.00, *P < 0.05 and N indicates not significant.

The cytotoxicity of the soft corals *L. arboreum, S. polydactyla*, *S. maxima* and *S. heterospiculata* were tested for the first time in U937 cells. In Figure 1 the cytotoxicity of extracts at 100 μg/ml is shown, indicating that these soft corals demonstrated potent cytotoxicity by killing more than 90% of the cells. Extracts of the mentioned organisms were significantly different (P < 0.000) from the values for negative controls (see Figure 1).

Moderate cytotoxicity (<60% of cell death) was demonstrated by the soft coral *S. trocheliophorum,* the green algae *C. prolifera,* the sponges *S. officinalis and H. rubens,* the sea grass *C. rondata* and the brown algae *T. turbinata.* The lowest cytotoxicity in U937 cells was observed from the mollusc *B. ampulla,* the jelly fish *C. andromeda* and the red algae *G. filamentosa* where 60% of the cells were still viable.

This is also the first report for the cytotoxicity of the mollusc *B. ampulla* and the soft coral *S. polydactyla* in HeLa cells. As shown in Figure 1, these organisms showed potent cytotoxicity by killing more than 90% of the cells when tested at 100 μg/ml. The soft corals *L. arboreum, S. maxima* and *S. trocheliophorum* showed strong cytotoxicity and killed more than 80% of the cells. *C. prolifera* and *S. heterospiculata* showed moderate toxicity (<60%). The lowest cytotoxicity in HeLa cells was observed for *S. officinalis, T. turbinata, C. andromeda, H. rubens* and *C rotundata* where more than 60% of the cells were still viable.

Following these observations only potent and the strongly active extracts were subjected to detailed study to determine their half maximal inhibitory concentrations (IC_50_) and selectivity indices. Once the IC_50_ values were determined, responses of the marine extracts were characterized according to the guidelines of the National Cancer Institute (NCI) where IC_50_ values of 10-20 μg/ml of a total/crude extract is viewed as cytotoxic, 20 ≤ 50 μg/ml as moderately cytotoxic and < 10 μg/ml as strongly cytotoxic. As the selectivity index value of an extract increase the ability of the extract to target the cancer cells rather than normal body cells also increases.

As shown in Table 1, the soft coral *L. arboreum* presented very strong cytotoxicity and selectivity (IC_50_; 6.5 μg/ml, SI; 6.45) in U937 cells which is reported here for the first time. It also showed a moderate activity in HeLa (IC_50_; 28.10 μg/ml, SI; 1.46) as compared to the cytotoxicity positive control, Actinomycin D (IC_50_ 7.8 μg/ml, SI 1.5) in U937 cells and (IC_50_; 17.7 μg/ml, SI; 1.5) in HeLa. The observed toxicity can be due to the terpenoids which are extensively reported in soft corals from the family Alcyoniidae [23]. Terpenoids, mainly macrocyclic cembrane-type diterpenoids and their derivatives, represent important chemical defense tools for these animals against their natural predators [24]. Results presented here are supported by current literature reporting that *L. arboreum* demonstrated moderate cytotoxicity against HUVEC, K-562 and HeLa [25], L-929 and K-562 [26] as well as strong antibacterial activity [27].

**Table 1:** **The half maximal inhibition concentration (IC**_**50**_**) of the lipophilic fraction of the marine organism extracts against HIV-1 PR is presented**

**Phylum**	**Marine organism**	**Cytotoxicity IC**_**50 **_**(μg/mL)**			**SI values**		**IC**_**50 **_**(μg/mL)**
		**U937**	**HeLa**	**Vero**	**U937**	**HeLa**	**HIV-1 PR**
Mollusca	*B. ampulla*	N/T^a^	76.53	110.80	N/A	1.4	N/T^b^
Rhodophyta	*G. filamentosa*	N/T^a^	N/T^a^	N/T^a^	N/A	N/A	2.6
Chlorophyta	*C. prolifera*	52.46	32.32	143.21	2.73	4.43	N/T^b^
Phaeophyta	*T. turbinatea*	N/T^a^	N/T^a^	N/T^a^	N/A	N/A	N/T^b^
Tracheophyta	*C. rotundata*	N/T^a^	N/T^a^	N/T^a^	N/A	N/A	N/T^b^
Porifera	*H. rubens*	N/T^a^	N/T^a^	N/T^a^	N/A	N/A	N/T^b^
Porifera	*S. officinalis*	N/T^a^	N/T^a^	N/T^a^	N/A	N/A	N/T^b^
Cnidaria	*C. andromeda*	N/T^a^	N/T^a^	N/T^a^	N/A	N/A	0.84
Cnidaria	*S. heterospiculata*	44.54	n/a	43.95	0.99	n/a	8.6
Cnidaria	*S. maxima*	24.00	30.08	43.95	1.83	1.46	13.1
Cnidaria	*S. polydactyla*	89.05	20.0	6.80	0.08	0.34	N/T^b^
Cnidaria	*L. arboreum*	6.50	28.10	41.90	6.45	1.46	12.0
Cnidaria	*S. trocheliophorum*	N/T^a^	5.200	10.90	N/A	2.10	N/T^b^
Actinomycin D^c^		7.8	17.7	11.8	1.5	0.7	N/T^b^
Acetylpepstatin^d^							5.7

The IC_50_ values were determined in different cell lines and are presented along with the associated selectivity index (SI) values which represent the relative activity towards the cancer cell lines compared to the Vero cells.

Not tested (N/T^a^) samples with inhibitory activities less than 50% at 100 μg/ml.

Not tested (N/T^b^) samples with inhibitory activities less than 80% at 100 μg/ml.

Superscript C indicates the anticancer drug used as positive control for the cytotoxicity assay.

Superscript D indicates the positive control used in the PR inhibition assay.

The soft coral, *Sarcophyton trocheliophorum* showed promising cytotoxicity with a selectivity index higher than the positive control (IC_50_ 5.2 μg/ml, SI; 2) in HeLa cells. In a similar study polyhydroxy sterols from *S. trocheliophorum* obtained in Singapore showed potent cell growth inhibitory activity against different human HL60 leukaemia (the present study used U937), M14 skin melanoma, and MCF7 breast carcinoma cells with EC_50_ values of 2.8, 4.3, and 4.9 μg/ml respectively detected by the MTT assay (a related, not identical tetrazolium dye was used here), and exhibited minimal toxicity to normal human peripheral blood lymphocytes [28]. These data suggests that *S. trocheliophorum* from any marine environment may be producing sterols which when isolated or present in an extract retain their potency and may yet prove useful as anti-cancer drugs.

Previous studies showed that a hybridization of *Sinularia polydactyla* and *Sinularia maxima* species had strong cytotoxicity in the following cell lines; the breast cancer SK-BR3 cell line, cervical cell line, HeLa and HeLa-Apl cell lines [29]. In the current study, when the original organisms (not hybrids) were tested, S*. polydactyla* exhibited moderate cytotoxicity (IC_50;_ 20 μg/ml, SI; 0.34) in HeLa cells. *S. maxima* had moderate toxicity (IC_50;_ 24.0 μg/ml, SI; 1.8) in U937 and in HeLa cells (IC_50;_ 30 μg/ml, SI; 1.46). In addition, another species that is reported here for the first time, *S. heterospiculata*, exhibited low cytotoxicity (IC_50;_ 44.5 μg/ml, SI_;_ 0.99) in U937 and no activity in HeLa cells.

The green algae *C. prolifera* showed weak to moderate cytotoxicity in U937 and HeLa cell lines (IC_50;_ 52.46 μg/ml, 32.3 μg/ml and SI_;_ 2.73, 4.4) respectively. Caulerpenyne (Cau), a metabolite from *Caulerpa taxifolia* has previously been shown to be cytotoxic against KB cells and fibroblasts from hamsters. Cau along with 6 other drugs representative of the major classes of anticancer products were tested against 8 cancer cell lines of human origin. Cau demonstrated growth-inhibitory effects in all cases with some variability between cell lines. It was also shown to induce inhibition of SK-N-SH tumour cell proliferation with an IC_50_ of 10 +/- 2 micro M [30-32]. It is there for possible that the cytoxicity demonstrated by the crude extract of *C.prolifera*, may increase if compounds like Cau is isolated from it. All other tested species showed moderate to weak activity against both cell lines.

The main goal of cancer chemotherapy research is to discover safe and efficient agents that target cancer cells specifically but are innocuous to normal cells. However, many anticancer drugs fail to meet this criterion being unable to discriminate between cancer and normal cells. The development of novel cancer chemotherapeutic agents with a higher potency and specificity against cancer cells is urgently needed. The selectivity index of *L. arboreum* in U937 and *S. trocheliophorum* in HeLa compares very well and even better than the positive control used in this study and is worthy of further investigation.

### Anti-HIV direct enzymes assays

All the extracts were tested at 100 μg/ml for the inhibition of HIV-1 RT and PR enzymes. The experiment was repeated at least 3 times. Extracts with percentages of inhibition ≥ 50% were considered to be active while the extracts demonstrating >90% inhibition were considered potent.

**Figure 2:**
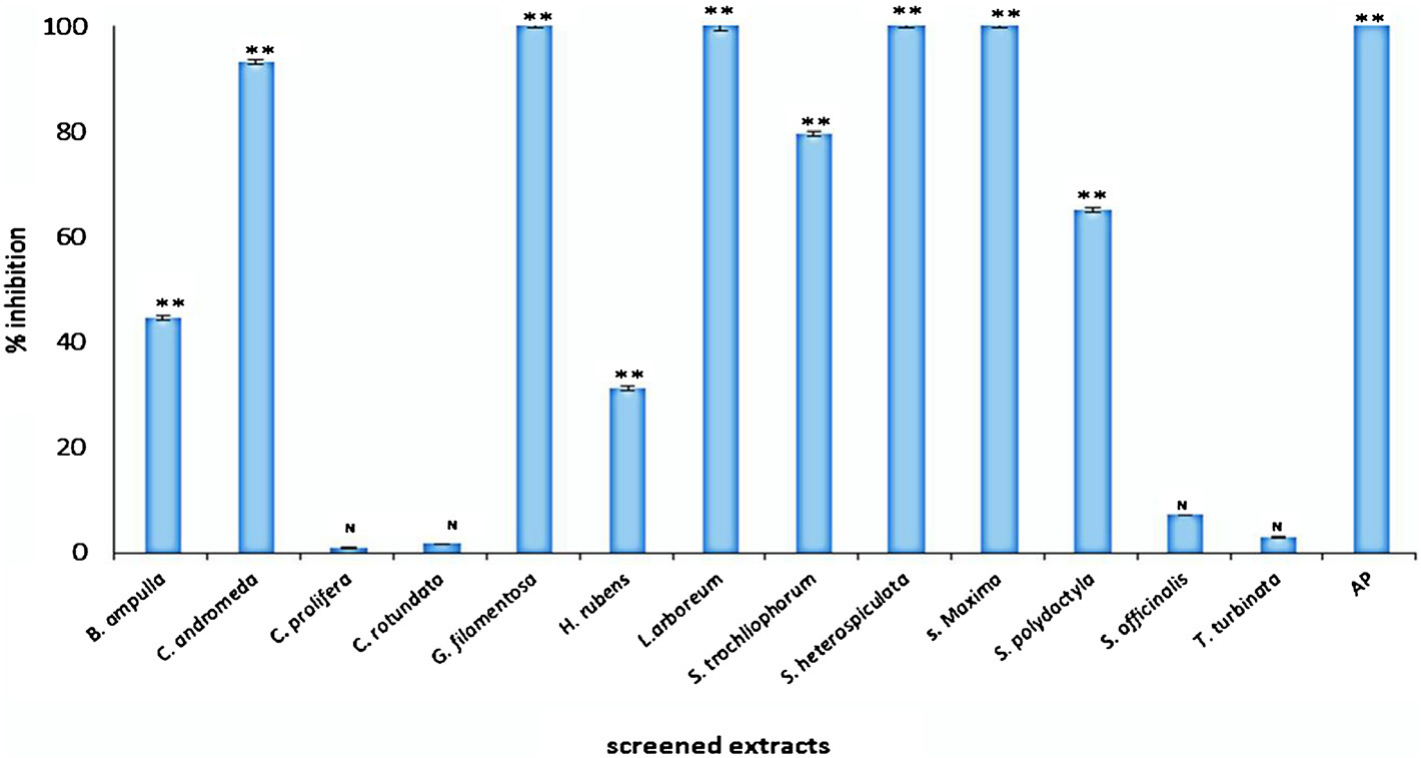
**HIV-1 PR inhibition percentages of different marine extracts.** Samples were dissolved in DMSO and diluted with the assay buffer to a final concentration of 100 μg/ml. Significant differences between the extracts and the DMSO control was calculated using IBM SPPS version 19. **P < 0.00, *P < 0.05 and N indicates not significant. AP indicates the experimental positive control acetyl pepstatin.

Unfortunately, none of the screened extracts showed any inhibitory activity of HIV-1 RT (all inhibitions were < 50%). In cases where marine organisms were able to inhibit this enzyme, it was always pure compounds which were hydrophilic in nature, suggesting that for activity against this enzyme, more aqueous extracts should be prepared [33]. On the other hand most of the extracts inhibited HIV-1 PR enzyme in the direct assays. These data is presented in Figure 2 demonstrating that strong inhibitory activity (>80%) were obtained by *S. maxima, L. arboreum*, *B. ampulla, C. andromeda, G. filamentosa*, and *S. heterospiculata*. Values of these active extracts were significantly different (P < 0.00) from the values for negative controls (See Figure 2). *S. trocheliophorum* and *S. polydactyla* was active but to a lesser extent demonstrating inhibitory activities of 65-80%. *C rotundata* and *C. prolifera* exhibited very weak inhibitory activity (< 50%).

Only the extracts with strong inhibitory activity were subjected to detailed study using serial dilutions of each extract in order to determine their IC_50_ values. Following IC50 assessments, the extract activities were classified as follows; potent activities were assigned to IC_50_ < 10 μg/ml, while IC_50_ 10 < 20 μg/ml was considered strong, and IC_50_ 20 < 50 μg/ml as moderate. Extracts demonstrating IC_50_ values of > 50 μg/ml were considered weak.

The detailed IC_50_ study showed potent inhibitory activity of HIV-1 PR by the Cnidarian jelly fish *C. andromeda* (IC_50_ 0.84 μg/ml). Phylum Cnidaria was reported to have antimicrobial [34], antioxidant [35] and anti-inflammatory [36] activities. Nothing has been reported on the anti HIV-1 or the cytotoxicity of *C. andromeda* until now.

In this study, the red algae *G. filamentosa* showed potent HIV-1 PR inhibitory activity (IC_50_ 2.6 μg/ml). Many studies have discussed the biological importance of marine algae. Some of the studies in search of anti-herpetic substances have been particularly interesting. The use of these organisms as botanical agents to treat viral infection resulted in four patents [37-39]. The first two patents used aqueous extracts of *Neodilsea americana* and *N. integra*[40] while the third patent is particularly well documented and involves the use of *Cryptosiphonia woodii*[41]. For the latter, clinical efficacy was clearly demonstrated. The fourth patent is for the use of carrageenan and other sulfated polysaccharides for the treatment of diseases (including AIDS) caused by retroviral infection [42]. In another study on the hydrophilic metabolites (sulfated oligo- or poly-saccharide compounds) from red algae, retroviral replication was suppressed and viral reverse transcriptase inhibited [33].

It is important to mention that our study is the first report of the promising HIV-1 protease inhibitory activity of *C. andromeda* (IC_50;_ 0.84) μg/ml and *G. filamentosa* (IC_50;_ 2.6 μg/ml) while the IC_50_ of the positive control (acetyl pepstatin) for enzyme inhibition in the assay was 5.7 μg/ml. These extracts showed no cytotoxicity against the three cell lines at the highest concentration (100 μg/ml) tested.

Moderate HIV-1 PR inhibitory activities were shown by the soft corals *S. heterospiculata* (8.6 μg/ml ±3.4), *L. arboreum* (12.007 μg/ml ±2.4) and *S. maxima* (13.1 μg/ml ± 2.3). Metabolites from soft corals found to have cytotoxicity against selected cancer cell lines, antiviral activity against human cytomegalo virus (HCMV), anti-inflammatory activity and antibacterial activity against five selected bacterial strains, have been reported [43]. To date, no anti-HIV activity have been reported in literature for the three soft corals tested here. Enzymatic assays are an important first step when identifying potential anti-HIV leads, however screening extracts and especially pure compounds in virus infected cell cultures are essential next steps to ensure that only the most promising of compounds able to inhibit whole virus replication progresses in the drug development pipe line.

## Conclusion

The present study demonstrated that marine organisms remain one of the most interesting sources for the discovery of bioactive leads that can help in the treatment of cancer and HIV. This study is the first to report the anticancer and anti HIV activities of *B. ampulla, C. rotundata, G. filamentosa, H. rubens, S. trochliophorum, S. heterospiculata, S. officinalis* and *T. turbinata.* This is also the first report on the HIV-1 PR inhibitory activities of extracts of *C. andromeda, C. prolifera* and *L. arboreum, S. maxima, S. polydactyla.*

Although the cytotoxicity of some of the extracts reported on here, have previously been tested in the HeLa cell line (for *C. prolifera*, *L. arboreum*, *S. maxima* and *S. polydactyla),* this study is the first to report cytotoxicity of these extracts in a leukaemia cancer cell line (U937).

The bio-activity evaluation of the extracts resulted in two marine extracts demonstrating strong anticancer activity; *L. arboreum* in U937 cells and *S. trochliophorum* in HeLa with high safety margins compared to Actinomycin D. *C. andromeda* showed potent anti-PR activity with an IC_50_ 6 times lower than the positive control with no cytotoxicity in the different cell lines while *G. filamentosa* extract showed double the activity of the positive control with higher safety margins. These findings suggest that these species could be an interesting source of bioactive compounds and deserve further bioassay-guided isolation procedures to determine the identity and structure of the active compounds.

## Abbreviations

HIV: Human Immunodeficiency Virus; PR: Protease; RT: Reverse transcriptase; FBS: Fetal bovine serum; IC50: The half maximal inhibitory concentration; SI: selectivity index; XTT: 2,3-bis-(2-methoxy-4-nitro- 5-sulfophenyl)-2H-tetrazolium-5-carboxanilide, Na2)

## Competing interests

The authors declare that they have no competing interests.

## Authors’ contributions

AH, DM and NL designed the study. AH and MSE was responsible for collection, identification and extraction of the samples; DM, NL and MSE performed the viability and the HIV enzymatic assays. MS wrote the first draft of the article, DM, AH; NL and MSE provided editorial and data interpretation information, all authors approved the final manuscript.

## Pre-publication history

The pre-publication history for this paper can be accessed here:

http://www.biomedcentral.com/1472-6882/14/77/prepub
